# Revolutionizing tomato disease detection in complex environments

**DOI:** 10.3389/fpls.2024.1409544

**Published:** 2024-09-16

**Authors:** Diye Xin, Tianqi Li

**Affiliations:** ^1^ East China University of Science and Technology, School of Information Science and Engineering, Shanghai, China; ^2^ East China University of Science and Technology, School of Biotechnology, Shanghai, China

**Keywords:** tomato leaf disease, Cascaded Group Attention, Real-Time-Detection-Transformer, lightweight backbone, feature fusion, Focaler-CIoU loss function

## Abstract

In the current agricultural landscape, a significant portion of tomato plants suffer from leaf diseases, posing a major challenge to manual detection due to the task’s extensive scope. Existing detection algorithms struggle to balance speed with accuracy, especially when identifying small-scale leaf diseases across diverse settings. Addressing this need, this study presents FCHF-DETR (Faster-Cascaded-attention-High-feature-fusion-Focaler Detection-Transformer), an innovative, high-precision, and lightweight detection algorithm based on RT-DETR-R18 (Real-Time-Detection-Transformer-ResNet18). The algorithm was developed using a carefully curated dataset of 3147 RGB images, showcasing tomato leaf diseases across a range of scenes and resolutions. FasterNet replaces ResNet18 in the algorithm’s backbone network, aimed at reducing the model’s size and improving memory efficiency. Additionally, replacing the conventional AIFI (Attention-based Intra-scale Feature Interaction) module with Cascaded Group Attention and the original CCFM (CNN-based Cross-scale Feature-fusion Module) module with HSFPN (High-Level Screening-feature Fusion Pyramid Networks) in the Efficient Hybrid Encoder significantly enhanced detection accuracy without greatly affecting efficiency. To tackle the challenge of identifying challenging samples, the Focaler-CIoU loss function was incorporated, refining the model’s performance throughout the dataset. Empirical results show that FCHF-DETR achieved 96.4% Precision, 96.7% Recall, 89.1% mAP (Mean Average Precision) 50-95 and 97.2% mAP50 on the test set, with a reduction of 9.2G in FLOPs (floating point of operations) and 3.6M in parameters. These findings clearly demonstrate that the proposed method improves detection accuracy and reduces computational complexity, addressing the dual challenges of precision and efficiency in tomato leaf disease detection.

## 1 Introduction

Tomatoes, rich in nutritional and medicinal value, are among the most significant crops cultivated globally. China ranks as a leading tomato producer globally (Coelho et al., 2023). In 2023, China, leveraging its vast agricultural landscape and favorable climate, solidified its status as the top tomato producer worldwide, contributing 67 million tons to the global total of approximately 190 million tons. This substantial output underscores China’s dominance in the global tomato market (Min, 2023). Moreover, China’s 2023 tomato production (Lu et al., 2023) exceeded initial forecasts, reaching 8 million tons, up from the predicted 7.3 million tons.

However, tomatoes face threats from various leaf diseases, including spot disease and leaf mold (Lee, 2022), caused by fungi, bacteria, and environmental stressors (Hernandez et al., 2021). Untimely detection and prevention can drastically reduce tomato yield and quality, resulting in significant economic losses for farmers.

Traditionally, tomato leaf disease detection has been manual, presenting numerous limitations and challenges. First, it depends on professional inspectors, leading to significant human resource constraints (Geisseler and Horwath, 2014). Second, factors like visual fatigue compromise the method’s accuracy. In large-scale settings like tomato plantations, manual detection becomes labor-intensive, increasing the risk of missed detections and false alarms (Lambooij et al., 2009). Consequently, automating tomato leaf detection has emerged as a key research focus to enhance efficiency and accuracy (Azim et al., 2014).

Advancements in computer technology have facilitated the incorporation of machine learning into agricultural research (Pallathadka et al., 2022)’s study preprocesses images with histogram equalization, followed by principal component analysis for feature extraction. Support vector machines and naive Bayesian classifiers are then employed for rice leaf disease classification. However (Sujatha et al., 2021), notes that machine learning’s extensive computational demands in preprocessing and feature extraction limit its practical application. Comparative studies have shown deep learning’s superior efficacy in plant leaf disease recognition, with convolutional neural networks (LeCun et al., 1998) and residual structures (He et al., 2016) leading to significant advancements in object detection algorithms, including the evolution to one-stage approaches like DETR with transformers. DETR (Detection Transformer) is an innovative object detection approach that utilizes transformers, which are originally designed for natural language processing tasks. By leveraging transformers, DETR simplifies the object detection pipeline, eliminating the need for hand-crafted components such as anchor generation and non-maximum suppression, and allows for direct end-to-end object detection with improved accuracy and efficiency.

Notably, two-stage models such as Faster RCNN (Region-based Convolutional Neural Network) (Ren et al., 2016) and Mask RCNN (He et al., 2017) have been typical (Teng et al., 2022). enhances pest detection with super-resolution modules (Dong et al., 2015) and Soft IoU (Rahman and Wang, 2016) mechanisms, achieving 67.4% accuracy on a pest dataset (Saleem et al., 2022). optimizes weed detection using Faster RCNN ResNet-101, with an enhanced anchor box method (Redmon and Farhadi, 2018) that refines region proposals and improves accuracy. RCNN3’s Mask RCNN-based algorithm (Wang et al., 2021) for crop images introduces path aggregation and feature enhancements (Liu et al., 2018), increasing edge accuracy with a micro fully connected layer (Lin et al., 2013). Despite these improvements, the large size, numerous parameters, and high computational costs challenge the practicality of two-stage algorithms.

Common one-stage algorithms encompass SSD (Single Shot MultiBox Detector) (Liu et al., 2016), YOLO v5(You-Only-Look-Once) (Jocher et al., 2022), YOLOv7 (Wang et al., 2023), and YOLOv9 (Wang et al., 2024) (Wang et al., 2022)’s YOLOv5 significantly enhances weed detection accuracy and speed via data augmentation (Simard et al., 2003) and converter encoder modules (Zhang et al., 2022). Experimental results indicate that the improved network surpasses the baseline YOLOv5 in F1 score, AP, and mAP@0.5 by 11.8%, 11.3%, and 5.9%, respectively (Zhang et al., 2023)’s study introduced a lightweight agricultural pest identification method using an enhanced Yolov5s, merged with MobileNetV3 (Howard et al., 2019), significantly lowering the network’s parameter count. Additionally, the study integrated the ECA (Efficient Channel Attention) attention (Wang et al., 2020) mechanism into MobileNetV3’s shallow network to boost performance. Experimental results reveal that compared to Yolov5s, their model cuts parameters by 80.3% with only a 0.8% drop in mAP, achieving a real-time detection speed of 15.2 FPS on embedded devices, outperforming the original model by 5.7 FPS.

The aforementioned one-stage algorithms have seen substantial optimization in speed and scale, yet their accuracy falls short of two-stage algorithms, rendering them less suited for high-precision applications in sectors like industry, agriculture, and emerging technologies (Agarwal et al., 2020). introduces a deep learning model with three convolutional layers and three max pooling layers for tomato leaf disease detection and classification. Outperforming established models like VGG (Visual Geometry Group)16, InceptionV3, and MobileNet, it achieves a classification accuracy of 91.2%. The study employs data augmentation and hyperparameter tuning to aid farmers in managing tomato diseases, enhancing crop yield and quality. Additionally, the DETR algorithm has shown significant accuracy in crop detection. The recent DETR (Carion et al., 2020) algorithm has also demonstrated notable accuracy in crop detection (Yang et al., 2023). introduces a DETR-based rice leaf disease detection algorithm, leveraging an enhanced detection transformer for diagnosis and recognition. Introducing the Neck structure and the Dense Higher Level Composition Feature Pyramid Network (Gao et al., 2019), based on FPN (Feature Pyramid Network), improves small disease target detection accuracy. However, DETR’s computational intensity, exacerbated by enhanced feature extraction, results in less favorable detection speeds and model parameters.

To facilitate a clearer understanding of the progress in this field, the methods utilized in the referenced literature are summarized in **Table 1**.

**Table 1:** Summary of detection methods for tomato leaf disease.

Method	Dataset	Train & Test	mAP50	FPS
Manual detection (Geisseler and Horwath, 2014)				
Automated detection technology (Azim et al., 2014)				
Support vector machines and Naive Bayesian classifiers (Pallathadka et al., 2022)	Rice Leaf Disease	Not mentioned		
Inception V3 (Sujatha et al., 2021)	Citrus leaf disease dataset	9:1	89.2	
Multi-Scale Super-Resolution RCNN (Teng et al., 2022)	Capured by Chinese Intelligent Machines Institute	8:2	67.4	
Enhanced Anchor Box-RCNN (Saleem et al., 2022)	DeepWeeds dataset	9:1	96.2	
Segmentation and Extraction Algorithm Based on Mask RCNN (Wang et al., 2021)	Fruit 360 dataset	9:1	94.9	
Real-time detection YOLOv5 (Wang et al., 2022)	Sugarbeet image dataset	9:1	90.0	20.8
Lightweight detection YOLOv5 (Zhang et al., 2023)	Large-scale open-source dataset IP102	9:1	98.6	15.2
CNN disease detection (Agarwal et al., 2020)	Tomato leaves dataset from plantvillage	20:1	91.2	
Dense Higher-Level Composition DETR (Yang et al., 2023)	IDADP dataset	Not mentioned	93.5	24.4

The motivation for developing the FCHF-DETR model arises from the serious economic losses and social impacts resulting from global crop diseases. Many farmers depend on the yield and quality of their crops for their livelihoods, and disease outbreaks not only threaten their food security, but can also inflict serious damage on the economic structure of entire regions.

In this context, the need for precise and timely disease detection is critical. The FCHF-DETR model employs advanced deep learning and real-time detection techniques to rapidly and precisely identify plant diseases in the field. This capability not only enables farmers to take timely measures to mitigate losses, but also offers a more stable and reliable management approach for agricultural production, thus effectively reducing the economic and social pressures arising from diseases.

Furthermore, the lightweight design of the FCHF-DETR model allows it to operate efficiently in resource-limited environments, critical for resource-poor agricultural areas. This design permits unrestricted model deployment across various hardware platforms, enabling farmers worldwide to utilize this technology and thereby enhance the sustainability and resilience of global agricultural production.

In summary, researchers have introduced numerous innovative methods and technologies in the field of object detection, which have significantly advanced the progress of plant disease management technology. To enhance applicability in crop production environments, this study introduces an accurate and lightweight tomato leaf disease detection model based on RT-DETR-R18. This model is characterized by its lightweight design, high detection accuracy, and rapid processing speed, facilitating easy deployment on farm detection equipment. The main contributions of this study include:

1. The integration of the lightweight and efficient Fasternet in lieu of the ResNet18 backbone network enhances the feature extraction speed by mitigating memory access and computational redundancy through the use of PConv (Partial Convolution) in Fasternet. This modification not only optimizes memory efficiency but also reduces the overall size of the model.
2. The substitution of the Attention-based Intra-scale Feature Interaction (AIFI) module with Cascaded Group Attention (CGA) within the Efficient Hybrid Encoder not only curtails computational expenditure but also enriches attention diversity. This is achieved by layering attention maps from different heads, facilitating a dual enhancement in both efficiency and accuracy.
3. The replacement of the High-Level Screening-feature Fusion Pyramid Networks (HSFPN) module with the CNN-based Cross-scale Feature-fusion Module (CCFM) module for inter-scale feature fusion within the Efficient Hybrid Encoder incorporates a channel attention mechanism. Given the dataset’s variety in terms of the types and sizes of diseased leaves, HSFPN adeptly assimilates global features across varying scales, synergizing with the decoder to accurately pinpoint locations.
4. Acknowledging the dataset’s heterogeneity and the varying levels of detection difficulty presented by diseased leaves, the model adopts the Focaler-IoU loss function in place of the conventional IoU loss. This strategic alteration aims at honing the focus on more challenging samples without amplifying the parameter count or computational complexity, thereby enhancing accuracy.

In the second section, we will delve into the dataset and the overarching architecture of FCHF-DETR. Moving on to the third section, we will undertake a series of ablation studies to dissect the impact of different modules on FCHF-DETR’s performance, complemented by visual illustrations. The fourth section is dedicated to a comparative analysis, highlighting the merits of our model vis-à-vis the prevalent RT-DETR-R18, and discussing prospective avenues for refinement. We will conclude by encapsulating the essence of our model and exploring its potential implications for practical applications.

## 2 Materials and methods

### 2.1 Data collection

**Figure 1:**
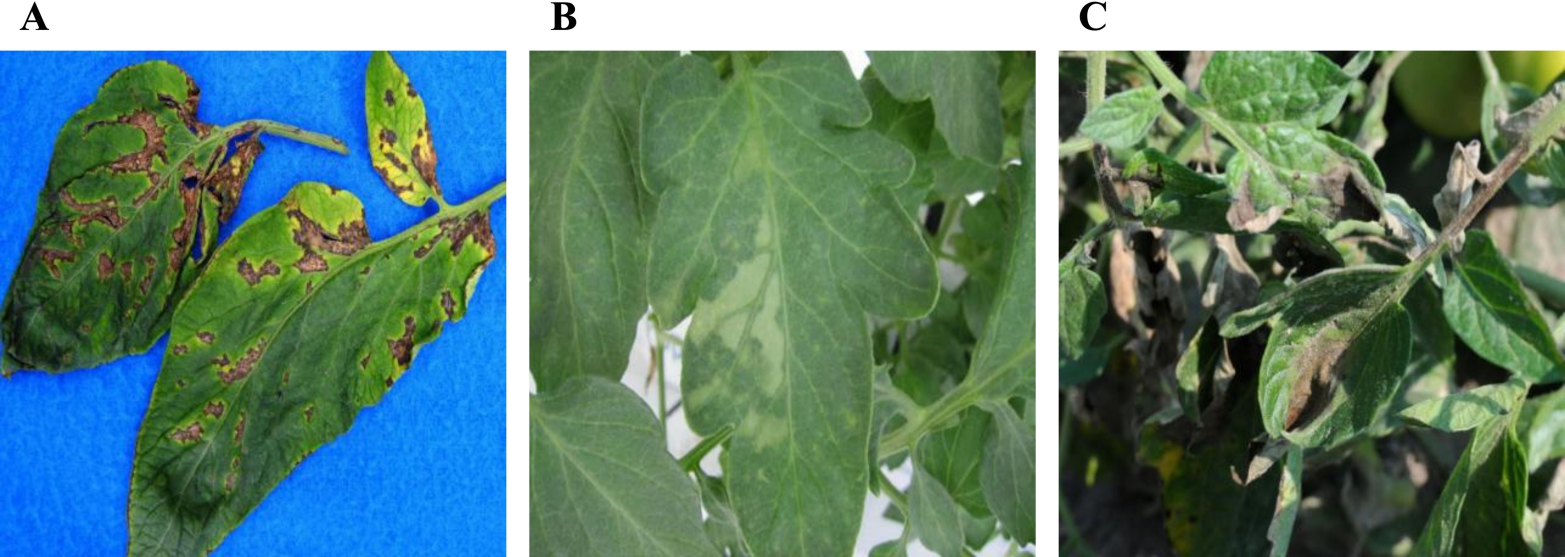
Samples of dataset, where **(A)** is the data from Tomato Leaf Diseases Detection Computer Vision dataset, **(B)** comes from Tomato Disease Multiple Sources dataset, **(C)** is the data collected for this paper’s research.

To improve the model’s generalization, the dataset includes tomato leaves photographed from multiple perspectives, backgrounds, lighting conditions, and featuring different disease types. A large collection of images was curated to enable accurate detection of minor diseases. However, due to the scarcity of public tomato leaf disease datasets, this study utilized the Tomato Leaf Diseases Detection Computer Vision dataset (**Figure 1A**) and the Tomato Disease Multiple Sources dataset (**Figure 1B**) from Kaggle. Despite their usefulness, these datasets have limitations, especially the oversimplified backgrounds with isolated leaves, which differ from real-world scenarios.

To overcome this and enhance the model’s ability to detect small-scale diseases, we augmented these datasets with 512 additional tomato leaf photos we collected (**Figure 1C**), creating a comprehensive dataset of 3147 images for this experiment. This carefully curated image collection features specimens of various resolutions and sizes, taken from many angles to ensure data diversity (**Figure 1**). The detailed presentation of tomato leaves closely mirrors actual detection settings, including the effects of natural elements like lighting and shadows. To simulate rainy-day detection conditions, we deliberately reduced the clarity of some images, emulating real-world challenges and enhancing the model’s robustness and applicability.

Images in the dataset were classified into five categories using LabelMe software: ‘Late blight leaf’, ‘Early blight leaf’, ‘Septoria leaf spot’, ‘Mold leaf’, and ‘Yellow virus leaf’. In the experimental setup, the dataset was divided into training, validation, and testing sets in an 8:1:1 ratio.

### 2.2 Date preprocessing

**Figure 2:**
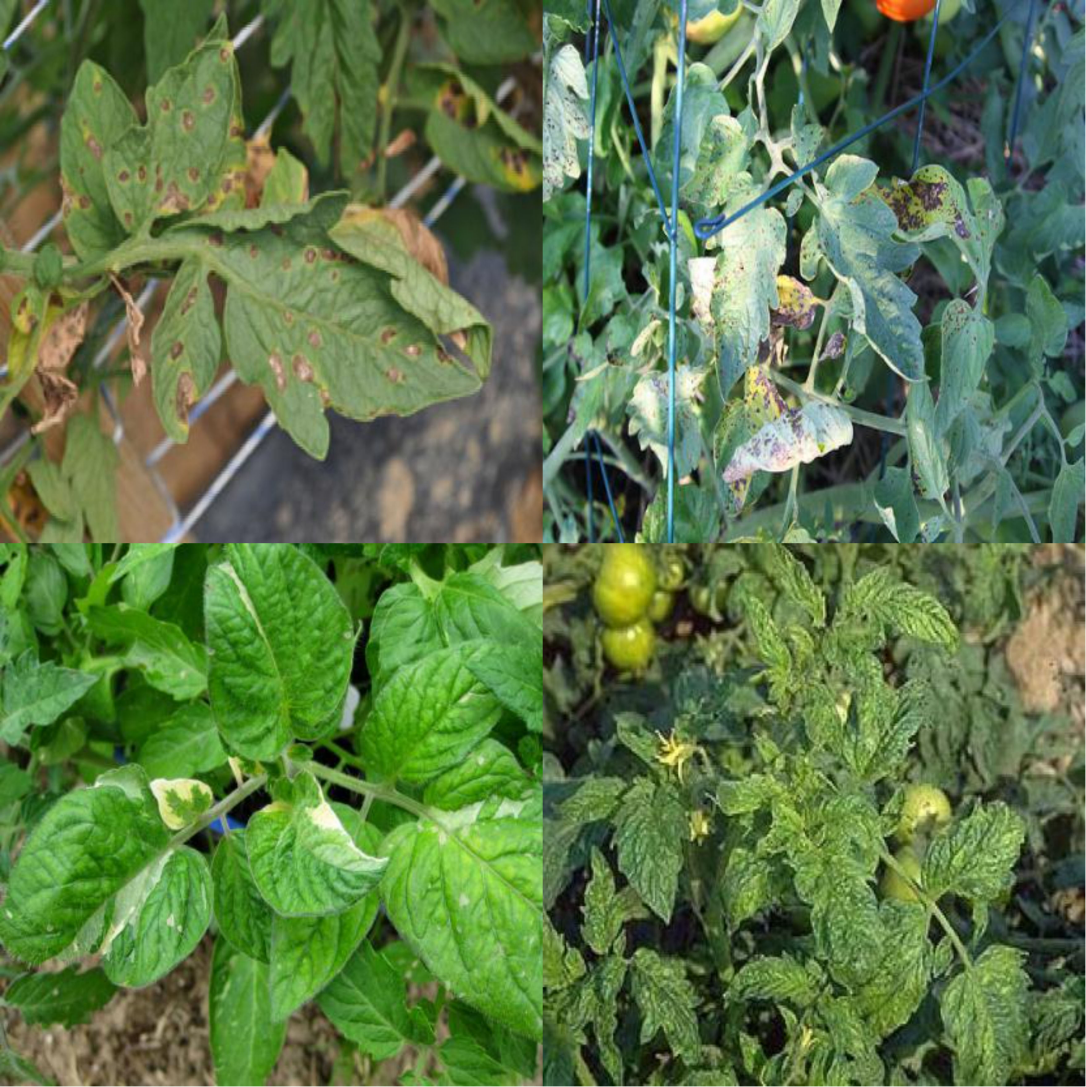
Mosaic data augmentation, randomly combining four pictures together.

During data preprocessing, we utilized the Mosaic data augmentation technique (Bochkovskiy et al., 2020) to combine four unique images into one composite image. This composite image undergoes random scaling, flipping, shifting, and color adjustments to enhance the model’s generalization ability. This technique enriches the dataset with extensive contextual details and various object instances in each synthesized image, as shown in **Figure 2**.

In tomato leaf disease detection, the uneven distribution of smaller target samples could hinder the model’s training efficiency. Using the Mosaic augmentation not only increases the sample volume but also balances the distribution of smaller targets, improving the model’s ability to detect them. Visualizing the disease targets and bounding boxes clarifies the spatial distribution of label centroids, with ‘x’ and ‘y’ axes representing the centroids’ coordinates and color intensity indicating proximity to the image center.

**Figure 3:**
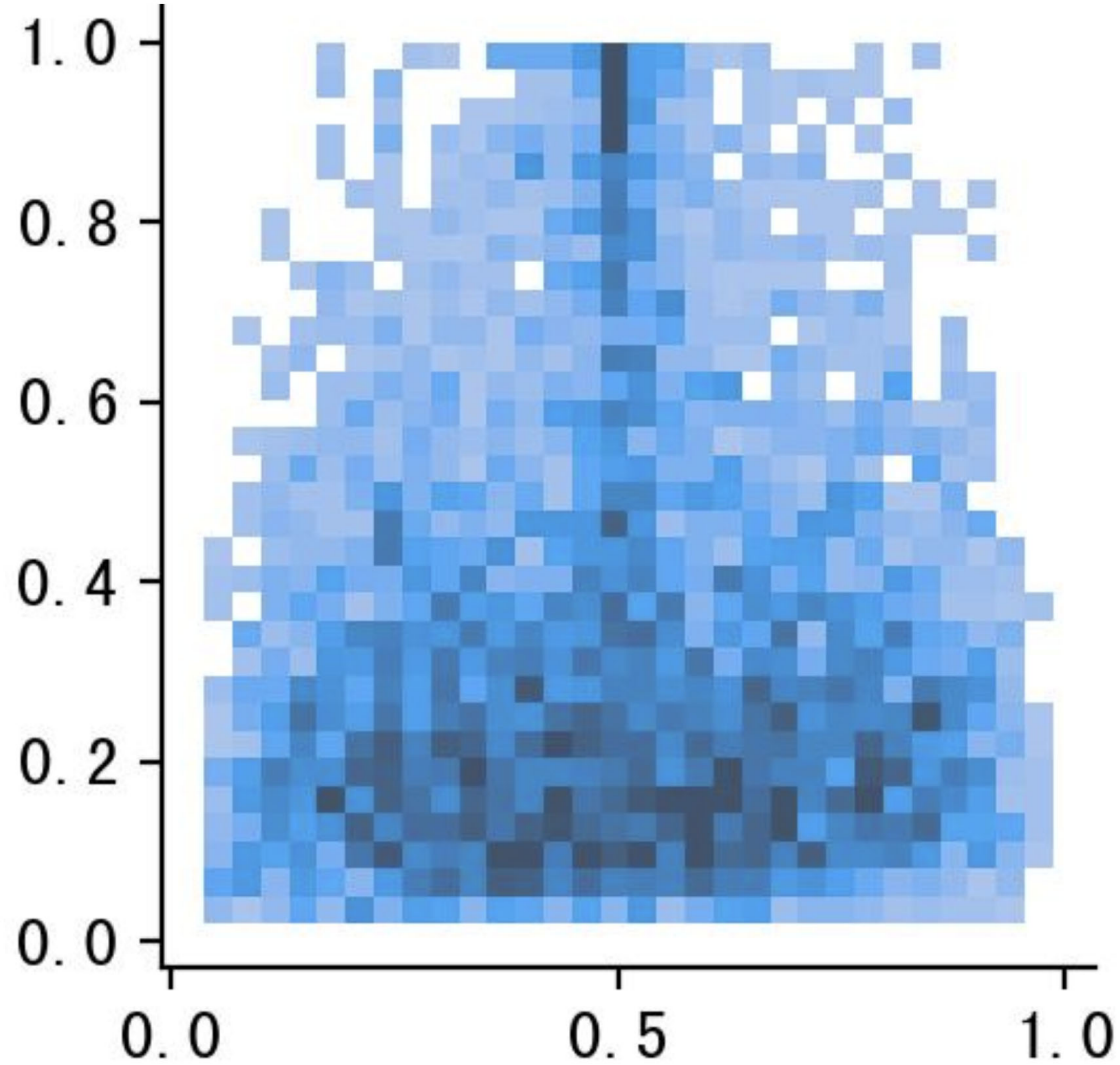
Tomato leaf disease size quantification framework based on target detection.

The visualization (**Figure 3**) highlights the distribution of target box sizes in the dataset, showing a relatively uniform color gradient across the image. This uniform color gradient suggests a balanced mix of large and small targets, achieved by careful preprocessing of the defect bounding boxes. This processing ensures fair representation of all target sizes in the dataset, counteracting any original bias towards larger defects. Aiming for a uniform distribution of defect sizes enhances the model’s ability to detect anomalies at various scales. This approach reduces size-related bias during training, enabling the model to accurately identify defects of different sizes in real scenarios. Ultimately, this preprocessing effort boosts the model’s generalization and balances its detection ability, leading to enhanced overall performance.

### 2.3 Overall structure of FCHF-DETR

**Figure 4:**
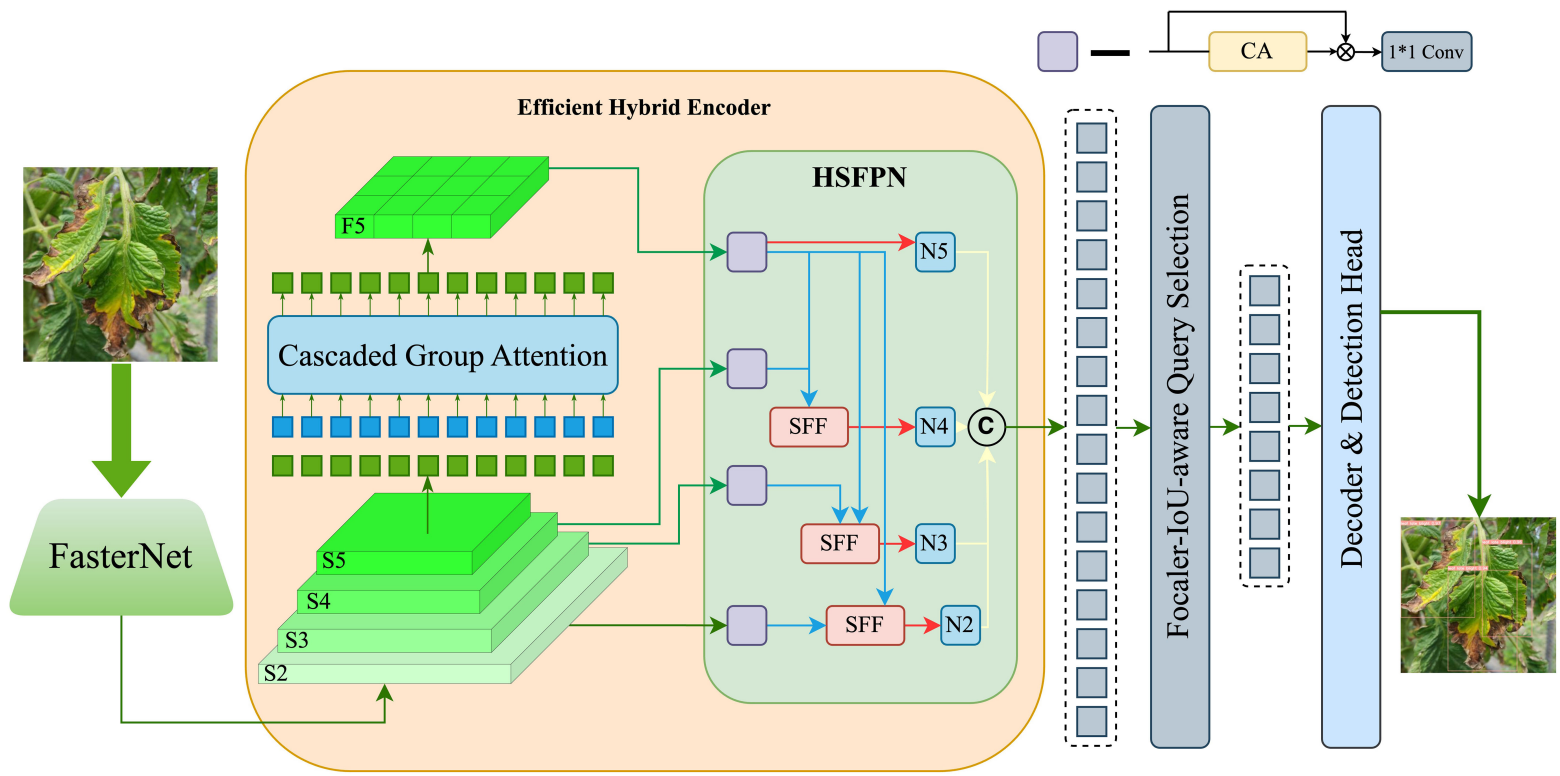
The overall architecture of FCHF-DETR, which contains Backbone, Cascaded group attention (CGA), High-level screening feature-fusion pyramid network (HSFPN), Focaler-CIoU loss function and Detection head.

This study presents the FCHF-DETR model (**Figure 4**), a streamlined yet precise detection network for various tomato leaf diseases, based on the RT-DETR-R18 (Lv et al., 2024) framework. The detailed structure of the proposed FCHF-DETR model is outlined below.

RT-DETR-R18 and the newly introduced FCHF-DETR are based on three main components: the Backbone, the Hybrid Encoder, and the Transformer Decoder. The Backbone acts as a feature extraction unit, effectively distilling multi-level features from input images, especially from the last three stages, S3, S4, and S5. These features are then fed into the Hybrid Encoder for further processing, which includes the AIFI module focusing on S5 feature maps to enhance precision and reduce complexity, and the CCFM module working with S3 and S4 features, using fusion blocks for feature amalgamation, refined by 1x1 convolutions.

RT-DETR-R18’s original backbone, based on ResNet18, contained numerous convolutional modules, hindering real-time detection and mobile deployment. Additionally, early versions of the AIFI module did not significantly improve accuracy. To address these challenges, this study introduces the FCHF-DETR approach, carefully crafted for efficient and accurate tomato leaf disease detection. Key improvements include integrating FasterNet instead of ResNet18 and adding PConv layers to enhance feature extraction speed and reduce model size; replacing the AIFI module with Cascaded Group Attention for increased efficiency; substituting the CCFM module with HSFPN for better feature fusion; and adopting the Focaler-IoU loss function to improve accuracy for difficult samples without increasing complexity.

#### 2.3.1 Lightweight network establishment

**Figure 5:**
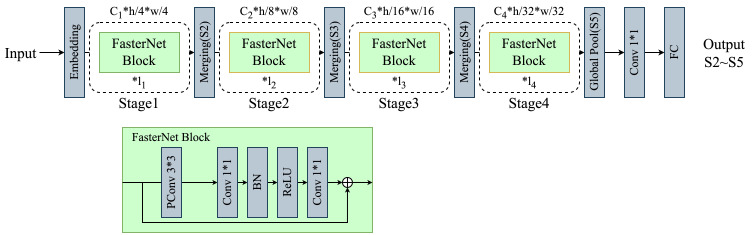
Fasternet’s backbone leverages deep learning for efficient feature extraction and accelerated neural network computations.

RT-DETR-R18’s ResNet-18 backbone, filled with convolutional modules, results in high computational needs and a large parameter count. Targeting mobile device deployment, this study prioritizes precise detection, faster inference, fewer parameters, and improved device compatibility. FCHF-DETR features a streamlined Backbone with FasterNet (Chen et al., 2023), balancing quick processing and accuracy, as depicted in **Figure 5**. FasterNet’s core includes FasterNet Blocks and PConv layers, dynamically adjusting convolution ranges based on data relevance for efficient processing.

##### 2.3.1.1 Partial convolution

**Figure 6:**
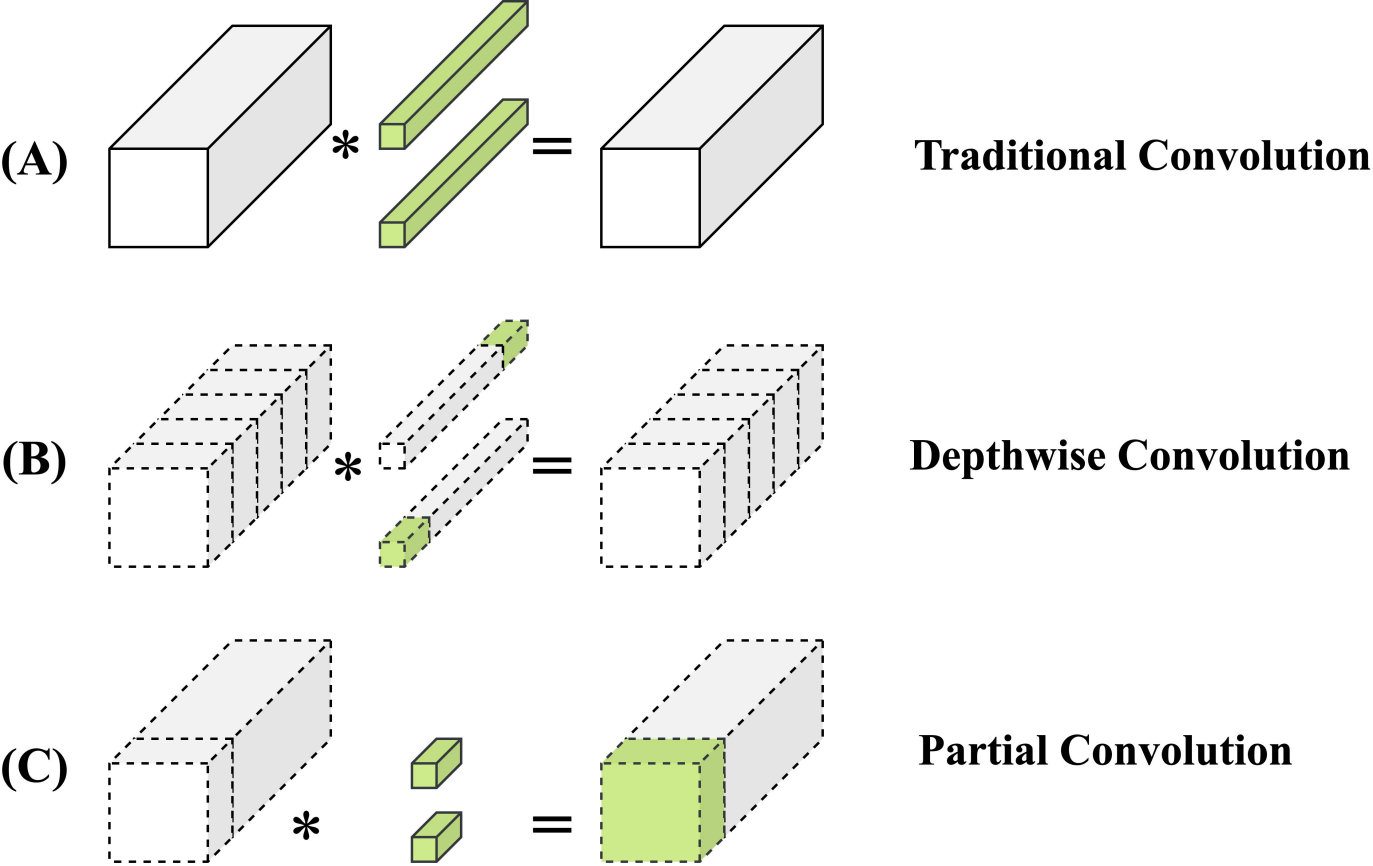
**(A)** Standard convolution applies filters across the entire input. **(B)** Depth-Wise convolution separates channels for independent processing. **(C)** Partial convolution dynamically adapts to missing data areas.

Partial convolution, or PConv, uses a unique binary masking technique to accurately distinguish valid from invalid data points. It dynamically adjusts the convolution kernel’s reach according to this distinction, focusing the convolution process on valid data. This method greatly enhances the model’s resilience in data incompleteness scenarios, preserving maximum information and minimizing data gap impacts. Compared to traditional convolutions (**Figure 6A**), PConv provides greater flexibility, efficiency, and precision in processing datasets with missing entries. Unlike Depth-Wise (**Figure 6B**) separable convolution (Chollet, 2017), known for fewer parameters and efficiency, PConv excels in managing complex imaging tasks with missing areas (**Figure 6C**). This suitability makes PConv ideal for applications like image restoration and content filling, where it effectively addresses image voids.

Given the similarity across feature maps of different channels, PConv efficiently performs convolution on a subset of input channels to extract spatial features, as shown in the **Figure 6C**. This method leaves the other channels unchanged. Assuming equal channel counts for input and output feature maps, PConv’s computational complexity, in terms of FLOPs, is significantly reduced:


$$FLOPs_{PConv}=h\times w\times k^{2}\times c_{p}^{2}$$

Where:

*h*, *w* are the width and height of the feature map,

*k* is the size of the convolution kernel,

$c_{p}$ is the number of channels for conventional convolution.

In practical implementation, there is generally $r=c_{p}/c=1/4$ , so the FLOPs of PConv are only 1/16 of those of conventional convolutions.

Memory access status of PConv:


$$MEM_{PConv}=h\times w\times 2c_{p}+k^{2}\times c_{p}^{2}\approx h\times w\times 2c_{p}$$

Where:

*h*, *w* are the width and height of the feature map,

*k* is the size of the convolution kernel,

$c_{p}$ is the number of channels for conventional convolution.

The memory access count of PConv is only 1/4 of that of regular convolution, and the remaining $\left(c-c_{p}\right)$ channels do not participate in the calculation, so there is no need for memory access.

RT-DETR-R18’s backbone network focuses on improving detection accuracy with a complex structure and more parameters for slightly enhanced capabilities. However, this approach may impact computational and memory efficiency. In fast-processing and resource-limited scenarios, like tomato leaf disease detection, FasterNet’s streamlined architecture could provide a better balance of accuracy and efficiency.

#### 2.3.2 Cascaded group attention

The attention mechanism is pivotal in tomato leaf disease recognition, with its primary capability being the substantial enhancement of recognition accuracy and processing efficiency through the focus on and emphasis of key features related to diseases in images. In environments characterized by complex backgrounds or varied disease manifestations, traditional image recognition techniques can overlook important details or result in misjudgments due to information overload. In contrast, the attention mechanism significantly improves the model’s effectiveness in distinguishing between healthy and diseased leaves through the construction of rich feature interactions and the optimization of importance allocation. This mechanism guarantees that the model maintains high recognition accuracy even amidst complex backgrounds or in cases of unclear symptoms.

**Figure 7:**
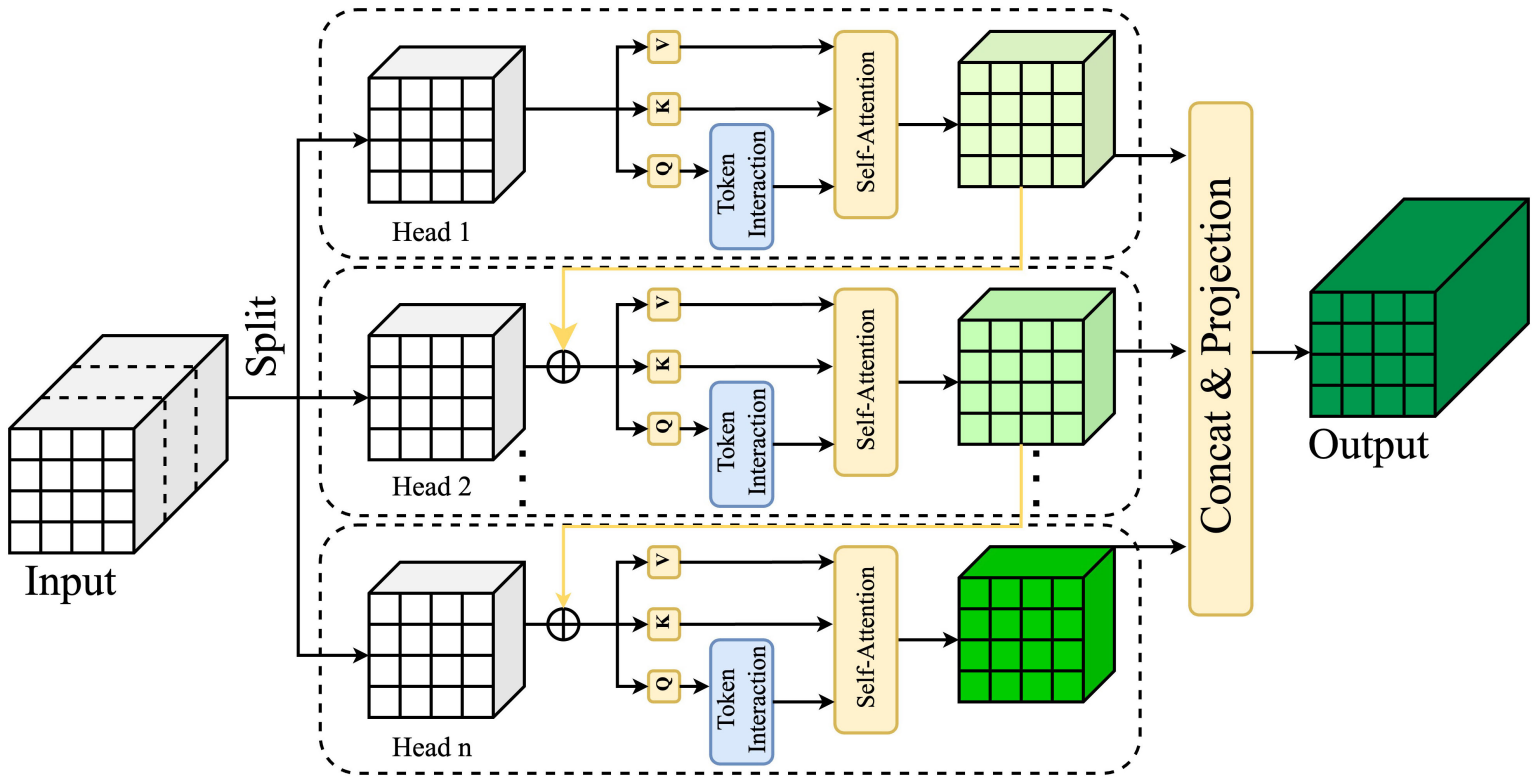
Cascaded Group Attention employs sequential attention layers, grouping features to focus progressively, enhancing representation by refining attention at multiple scales for improved contextual learning.


We’ve incorporated the Cascaded Group Attention (CGA) (Chen et al., 2023) mechanism, shown in **Figure 7**, to effectively address the computational efficiency challenges often found with the SE attention (Hu et al., 2018) approach. Traditional mechanisms such as SimAM (Yang et al., 2021) falter in complex scenes, and CBAM’s (Woo et al., 2018) complexity may overload the model, slowing down inference. Unlike SE, CA, and CBAM, CGA excels in nuanced feature processing via systematic grading and grouping, enhancing feature differentiation. CGA highlights inter-channel and spatial relationships and uses a cascaded framework to enrich layers with informative attention outputs. This progressive approach makes CGA highly adaptable and effective in managing complex features, providing a balanced depth and breadth in analysis.

$${\tilde{x}}_{ij}=Attn\left(X_{ij}W_{ij}^{Q},X_{ij}W_{ij}^{K},X_{ij}W_{ij}^{V}\right)$$

$${\tilde{x}}_{i+1}=Concat{\left[{\tilde{x}}_{i,j}\right]}_{j=1:h}W_{i}^{p}$$

Where:

*j*-th head computes the self-attention over $X_{ij}$ represents the *j*-th split of the input feature $X_{i},\;i.e.$ ,

$X_{i}=\left[X_{i1},\;X_{i2},\ldots \ldots ,X_{ih}\right]$ and 1≤j≤h , *h* represents the total number of heads,

$W_{ij}^{Q}$ , $W_{ij}^{K}$ , $W_{ij}^{V}$ represent projection layers mapping the input feature split into different subspaces,

$W_{i}^{P}$ represents a linear layer that projects the concatenated output features back to the dimension consistent with the input.

Using feature segmentation instead of the full feature set for each attention head is more efficient and reduces computational cost. While effective, this approach can be improved by enabling the Q, K, and V layers to project richer features, thus enhancing their capabilities. A cascading strategy for attention maps, as shown in the **Figure 7**, involves incrementally adding each head’s output to the next, enhancing feature refinement. This systematic accumulation enables progressive refinement of feature representation:


$$X_{ij}^{'}=X_{ij}+{\tilde{X}}_{i\left(j-1\right)},\;1<j\leq h$$

Where:

$X_{ij}^{'}$ represents the addition of the j−th input split $X_{ij}$ and the (j−1) -th head output ${\tilde{X}}_{i\left(j-1\right)}$ .

In the self-attention computation, we redefine $X_{ij}$ as the novel input feature for the j−th attention head. Furthermore, we’ve introduced an additional Token Interaction layer post Q-projection, enriching the self-attention mechanism’s capability to concurrently apprehend local and global relationships, thereby amplifying the feature representation.

In our work, we replaced RT-DETR-R18’s original AIFI module with the CGA approach, yielding two key advantages. Firstly, varied feature segmentation for each head enhances attention map diversity. This is similar to group convolution, where cascaded group attention can save Flops and parameters by a factor of h. Secondly, layering the attention heads deepens the network, enhancing capacity without additional parameters. With reduced channel dimensions for Q and K in attention map computations, the resulting latency overhead is minimal. This refined approach enables precise disease localization across sizes in tomato leaf disease detection, significantly improving detection accuracy.

#### 2.3.3 High-Level Screening-feature Fusion Pyramid Networks

**Figure 8:**
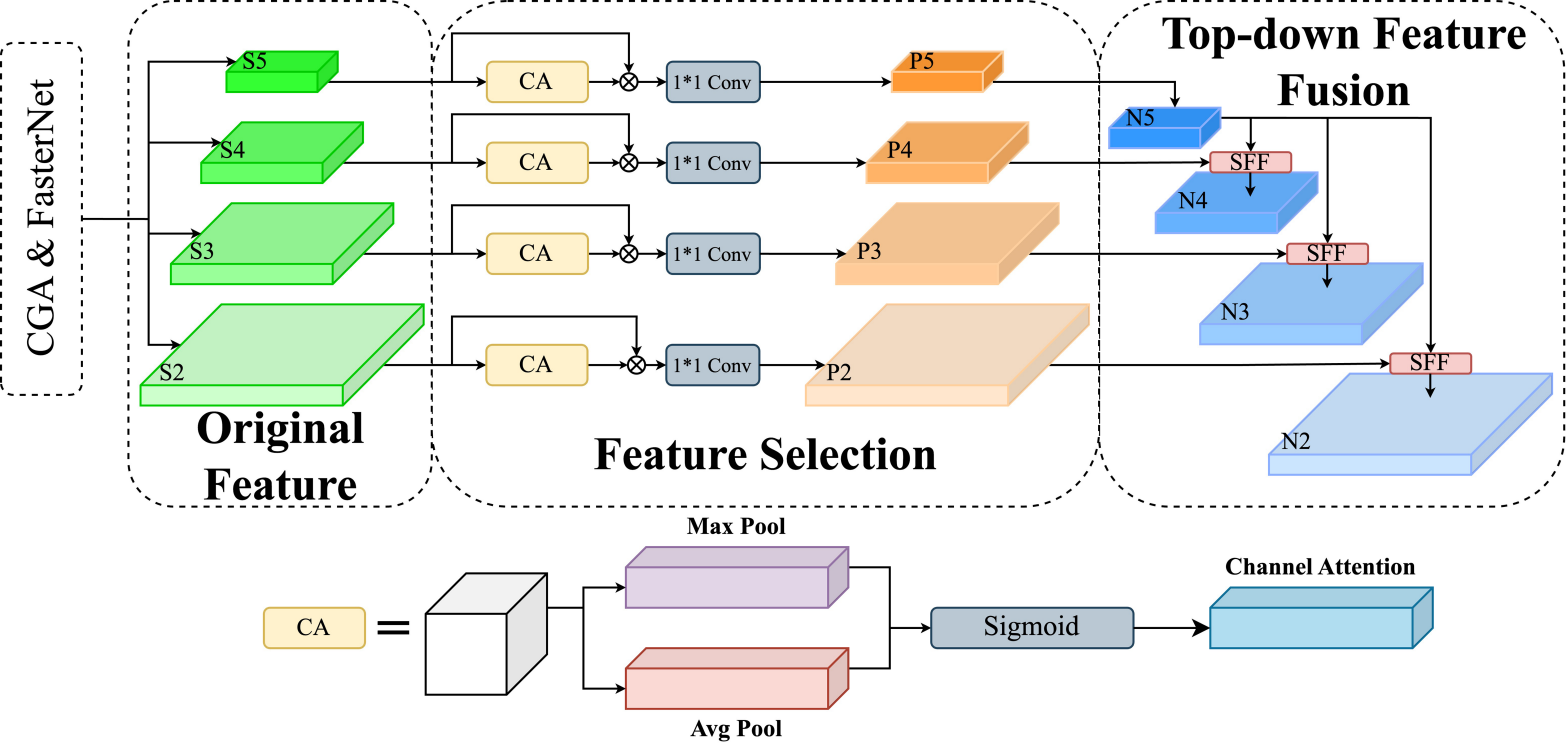
High-Level Screening-Feature Fusion Pyramid Networks (HSFPN) integrate multi-scale features with high-level screening for enhanced object detection, achieving superior performance through hierarchical feature fusion.

The High-Level Screening-feature Fusion Pyramid Network (HSFPN) (Chen et al., 2024) is crafted to build hierarchical feature pyramids attuned to scale variations, as shown in **Figure 8**. This design allows HSFPN to precisely detect disease features on tomato leaves, varying in size and shape, thus improving detection accuracy and robustness. Furthermore, HSFPN’s layered approach to feature fusion preserves detailed information, crucial for identifying early-stage or subtle leaf disease indicators. Consequently, HSFPN outperforms CCFM, particularly in complex agricultural settings and in detecting finely detailed objects.

##### 2.3.3.1 Selective Feature Fusion

**Figure 9:**
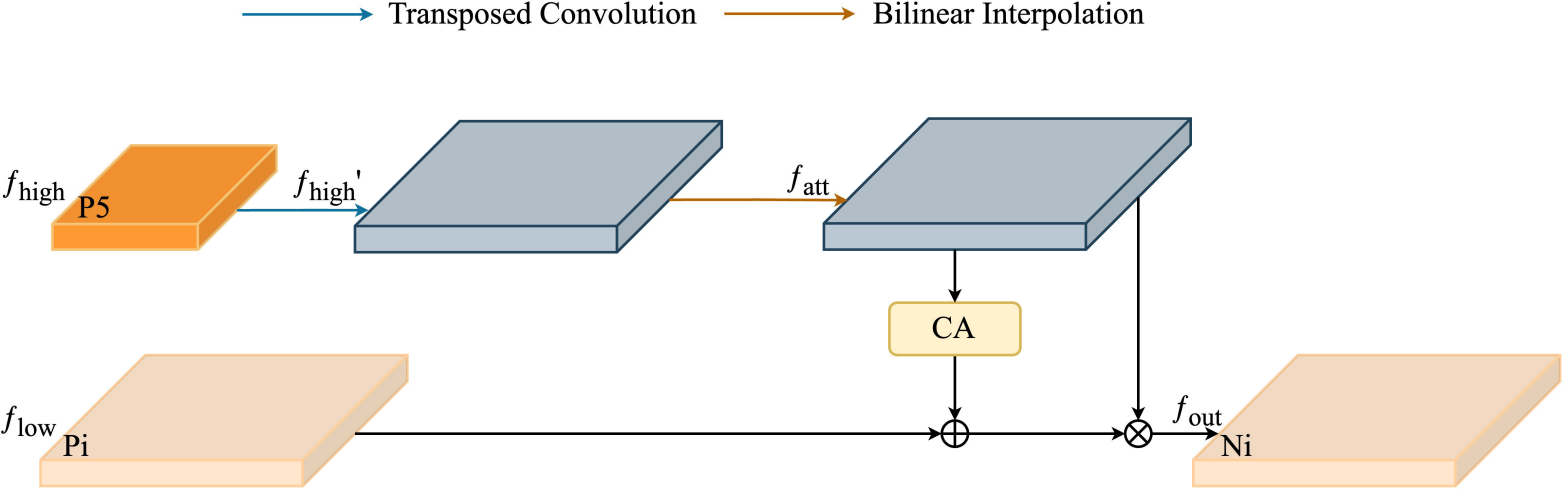
Selective feature fusion in High-Level Screening-Feature Fusion Pyramid Networks intelligently merges critical high-level features, enhancing object detection by optimizing feature representation.

Selective Feature Fusion (SFF), key to HSFPN, shown in **Figure 9**, crucially combines feature maps from various scales. The SFF module uses higher-level features as weights to filter through and selectively extract relevant information from low-level features. This involves scaling higher-level features to match low-level feature dimensions, using methods like transposed convolution and bilinear interpolation. Then, these scaled higher-level features act as attention weights to highlight valuable insights from low-level features. This fusion strategy effectively combines the semantic depth of high-level features with the detailed nuances of low-level features, greatly improving the model’s ability to handle multi-scale data challenges.

Given a high-level feature $f_{high}\in R^{C*H*W}$ and a low-level feature $f_{low}\in R^{C*H_{1}*W_{1}}$ , the process begins by expanding $f_{high}$ through a transposed convolution operation. This operation utilizes a stride of 2 and a kernel size of 3×3 , enlarging $f_{high}$ to a new dimension $R^{C*2H*2W}$ .

Following this, to reconcile the dimensions of the high-level and low-level features, bilinear interpolation is employed to either upscale or downscale the high-level features. This adjustment results in a feature $f_{att}$ that matches the low-level feature dimensions in $R^{C*H_{1}*W_{1}}$ , thus facilitating their subsequent integration:


$$f_{att}=BL\left(T-Conv\left(f_{high}\right)\right)$$

$$f_{att}=f_{low}*CA\left(f_{att}\right)+f_{att}$$

Next, use the CA module to convert advanced features into corresponding attention weights to filter out low-level features, after obtaining features with the same dimension. Finally, the filtered low-level features are fused with high-level features to enhance the feature representation of the model and obtain $f_{out}\in R^{C*H_{1}*W_{1}}$ .

Integrating HSFPN with CCFM significantly enhances disease detection precision in tomato leaf images, especially for size-varying disease manifestations. HSFPN’s layered feature pyramid architecture skillfully captures and defines features across scales, greatly improving the model’s sensitivity and accuracy in identifying disease stages, from small lesions to widespread areas.

HSFPN’s strategic use of multi-scale features not only strengthens the model’s ability to detect small targets but also maintains accuracy for larger ones. This dual strength effectively addresses traditional challenges in detecting varying disease sizes, offering robust support for precision agriculture’s complex requirements.

#### 2.3.4 Focaler-CIoU

Sample imbalance is a common issue in object detection, typically appearing as simple and difficult samples, categorized by target size. Simple samples involve easier-to-detect targets, while difficult samples include very small targets, challenging accurate localization.

In tasks with mainly simple samples, focusing on bounding box regression for these targets can significantly improve detection. Conversely, in scenarios with prevalent difficult samples, refining regression for these targets becomes essential. To address this variance, the IoU loss function can be adapted using a linear interval mapping method (Zhang and Zhang, 2024). This method enables flexible adjustment between simple and difficult samples, fine-tuning bounding box regression accuracy and improving detection performance. The modified IoU loss function, designed to address sample imbalance, is mathematically defined as follows:


$$IoU=\frac{\left|B\cap B^{gt}\right|}{\left|B\cup B^{gt}\right|}$$

$$IoU^{focaler}=\{\begin{array}{c} 0,\;IoU<d\\ \frac{IoU-d}{u-d},\;d\ll IoU\ll u\;\;\;\;\;\;\;\;\;\;\;\;\;\;\;\;\\ 1,\;IoU>u \end{array}$$

Where:

*B* represents the predicted box

$B^{gt}$ represents the GT (goal target) box

$IoU^{focaler}$ is the reconstructed Focaler-IoU

IoU is the original IoU value


[d,u]∈[0,1]

Applying Focaler-IoU loss to existing IoU based bounding box regression loss function CIoU:


$$CIoU=IoU-\frac{\rho^{2}\left(b,b^{gt}\right)}{c^{2}}-\alpha \upsilon$$

$$\alpha =\frac{\upsilon }{\left(1-IoU\right)+\upsilon }$$

$$\upsilon =\frac{4}{\pi^{2}}{\left(arctan\frac{w^{gt}}{h^{gt}}-arctan\frac{w}{h}\right)}^{2}$$

$$L_{Focaler-CIoU}=L_{CIoU}+IoU-IoU^{Focaler}$$

Where:

*b* represents the center points of anchor box

$b^{gt}$ represents the center points of GT box

ρ(⋅) represents the Euclidean distance

*c* represents the diagonal minimum distance enclosing bounding box between *b* and $b^{gt}$

$w^{gt}$ represents the width of GT box

$h^{gt}$ represents the height of GT box

*w* represents the width of anchor box

*h* represents the height of anchor box

In the field of tomato leaf disease detection, the Focaler-CIoU loss function offers significant advantages over the loss function originally used in RTDETR. Focaler-CIoU enhances the model’s ability to recognize challenging samples by adjusting the loss function to focus on samples of varying difficulty levels, particularly for disease samples that are challenging to distinguish or have indistinct boundaries, by assigning higher weights. This is particularly important when dealing with lesions of varied sizes and shapes on tomato leaves, as accurately identifying these diseases in their early stages is often challenging. The characteristic of Focaler-CIoU can significantly enhance the sensitivity in detecting early or minor lesions, lower the rate of missed detections, and thus boost the overall detection efficiency while maintaining high accuracy. It holds considerable importance in enhancing the early prevention and control of tomato leaf diseases.

## 3 Results

This section details the experimental, hyperparameter settings, and training strategies in Section 3.1. Section 3.2 describes the indicators and calculation formulas employed to evaluate model performance. Sections 3.3 and 3.4 discuss the study’s results, utilizing ablation experiments and visual displays, respectively.

### 3.1 Experimental setup

The experiment utilized an OpenBayes cloud server equipped with an Nvidia A100 80GB MIG 1g.10g graphics card, boasting 16GB of graphics memory, and ran on a Linux operating system. This experiment was implemented using Python 3.10 and Cuda11.8.

The model training strategy entailed: For IoU-aware query selection, the first 300 encoder features were selected to initialize the decoder’s object query. Training employed the AdamW optimizer, with a base learning rate of 0.0001, weight decay of 0.0001, global gradient clipping norm of 0.0001, 2000 linear warm-up steps, and spanned 100 epochs.

### 3.2 Evaluation indicators

In the field of object detection, performance is primarily evaluated by Precision (P), Recall (R), and Mean Average Precision (mAP). Precision represents the ratio of correctly predicted positive samples to all samples labeled as positive by the model. Recall measures the proportion of correctly identified positive samples among all actual positive samples. mAP denotes the mean of the average precisions across all categories. The corresponding formulas for Recall, Precision, and mAP are provided below:


$$P=\frac{TP}{TP+FP}$$

$$R=\frac{TP}{TP+FN}$$

$$AP=\int_{0}^{1}P\left(R\right)dR$$

$$mAP=\frac{\sum_{i=1}^{n}AP_{i}}{n}$$

TP (True Positive) refers to correctly identified positives, FN (False Negative) to positives incorrectly labeled as negatives, and FP (False Positive) to negatives incorrectly labeled as positives. Precision (P) is the ratio of correctly predicted positive observations to the total predicted positives, while Recall (R) is the ratio of correctly predicted positive observations to all actual positives. The area under the curve drawn through Precision (P) and Recall (R) values on the PR graph represents the Average Precision (AP), and the mean of AP values across all categories yields the Mean Average Precision (mAP).

Beyond the aforementioned performance metrics, model size and computational cost are assessed using the number of parameters and FLOPs, to facilitate the selection of a lightweight network for deployment on mobile devices. A reduction in parameters and FLOPs enhances model efficiency under identical computational resources, concurrently minimizing memory consumption and boosting computational speed.

### 3.3 Ablation experiment

Each module within FCHF-DETR was evaluated through ablation experiments to discern which modules enhance detection performance and which reduce computational and parameter costs. RT-DETR-R18 served as the benchmark model, with the introduction of the lightweight network structure, FasterNet, as FCHF-DETR’s backbone to assess its capacity to reduce model parameters and enhance inference speed effectively. Subsequently, the AIFI module in the Efficient Hybrid Encoder was replaced with Cascaded Group Attention to extract finer features. Additionally, the CCFM module was substituted with HSFPN, capable of capturing and expressing multi-scale features, thereby enhancing network accuracy. Ultimately, the model’s original loss function was optimized to the Focaler-CIoU loss function, adept at efficiently capturing edge information of tomato leaf diseases.

Initially, we evaluated the impact of integrating lightweight backbone networks versus not integrating them on the test set. Comparison of the benchmark model RT-DETR-R18 with RT-DETR FasterNet (Experiments 1 and 2) was performed. The introduction of lightweight backbone networks led to decreases of 1.9% and 0.5% in Precision and Recall, respectively. The mAP50-95 and mAP50 values decreased by 0.6% and 0.3%, respectively, while the number of Parameters decreased by 21%, the FPS increased by 1.8, and the FLOP decreased by 13.6%. These results suggest that FasterNet, as the backbone network of RT-DETR-R18, effectively reduces computational complexity and parameter count, and significantly enhances inference speed. Although the accuracy has marginally decreased, the improvement in efficiency renders this loss acceptable.

A lightweight network structure significantly trims model size and elevates detection speed, albeit at the expense of detection accuracy. Consequently, methods that enhance accuracy without incurring substantial computational costs are crucial.

Subsequently, employing the lightweight RT-DETR model with FasterNet as the backbone, we examined the performance alterations resulting from the integration of various modules. Experiments 3, 4, and 5 involved the replacement of the AIFI module in the original Efficient Hybrid Encoder with the SimAM, SE, and CGA attention mechanisms, respectively, each contributing to an improvement in accuracy. However, given the focus on lightweight networks in this study, the CGA attention mechanism was selected for further investigation. In Experiments 6 and 7, the CCFM module in the Efficient Hybrid Encoder was replaced by HSFPN without the SFF module and HSFPN with the SFF module, respectively. Upon comparison, the HSFPN with the SFF module, which offered greater accuracy improvements, was chosen. Building on Experiment 7, the loss function of the benchmark RT-DETR-R18 model was optimized, with both CIoU and Focaler-CIoU loss functions being employed for training. **Table 2** illustrates the enhancement in detection performance attributable to the lightweight DETR model.

- Experiments 3, 4, and 5 evaluated the integration of SimAM, SE, and CGA attention mechanisms, respectively, into the RT-DETR-R18 model with FasterNet as the backbone network. Compared to Experiment 2, the additions of SimAM, SE, and CGA resulted in increases of 0.9%, 1.8%, and 2.3% in the mAP50-95 index, respectively, and changes of -0.1%, 0.4%, and 0.7% in the mAP50 index for SimAM, SE, and CGA, respectively. The performance metrics suggest that SimAM, likely a non-parametric attention mechanism, notably improved the model’s size and inference speed. However, given that SimAM only slightly improved, or even reduced, accuracy, despite a comprehensive comparison, the CGA attention mechanism was ultimately selected due to its significant accuracy improvements, despite a slight increase in model parameters. Additionally, substituting the AIFI module with the selected attention mechanism enhanced the accuracy of tomato leaf disease detection, albeit with a minor reduction in inference speed and a slight increase in model parameters, aligning with the initial objective of replacing the AIFI module.
- Experiments 6 and 7 demonstrate that replacing the CCFM module in the RT-DETR model with HSFPN and HSFPN_SFF leads to significant improvements in the detection accuracy of the model. In the test set, HSFPN and HSFPN_SFF increased the parameter count by 0.3M and 0.5M, respectively, and reduced inference speed by 0.3 and 0.4, respectively. In Experiment 6, incorporating the HSFPN module yielded a 7% increase in Precision, a 1% increase in mAP50-95, and a 0.5G reduction in FLOPs. However, considering the increase in model parameters and the decrease in inference speed, the improvement in detection accuracy is deemed insufficient. In Experiment 7, the integration of the SFF module into feature fusion resulted in increases of 1% in P, 1.3% in Recall, 2.6% in mAP50-95, and 0.3% in mAP50. Although the model parameters have increased slightly and the inference speed is slower compared to FasterNet+CGA in Experiment 5, the significant improvement in detection accuracy relative to the benchmark network satisfies the lightweight standard.
- In Experiments 8 and 9, the loss functions of the benchmark network were substituted with CIoU and Focaler CIoU, respectively. Although the impact on inference speed, parameter count, and computational complexity is minimal, the CIoU loss function fails to yield a significant improvement in detection accuracy. However, optimization of the Focaler CIoU loss function led to increases of 0.3% in Precision and Recall, and 1.7% and 0.3% in mAP50-95 and mAP50, respectively. The uneven distribution of tomato leaf disease and the presence of small or edge targets in the images pose challenges to the detection capabilities of the model, which is expected. The introduction of the Focaler CIoU loss function significantly enhances the localization and detection of challenging targets, thereby enhancing the accuracy and robustness of the model for small, overlapping, and edge targets.

**Table 2:** Ablation experiment results: comparative analysis of all modules used in FCHF-DETR.

	Model	P	R	mAP50-95	mAP50	Parameters	FPS	GFLOPs
1	RTDETR-R18	94.7	93.6	83.1	96.2	19,880,748	21.9	57.0
2	RTDETR-FasterNet	92.8	93.1	82.5	95.9	15,792,928	23.7	49.5
3	RTDETR-FasterNet-SimAM	94.1	93.7	83.4	95.8	15,621,884	24.8	47.3
4	RTDETR-FasterNet-SE	94.9	95.2	84.3	96.3	16,882,972	21.7	54.5
5	RTDETR-FasterNet-CGA	95.1	95.1	84.8	96.6	15,812,212	24.5	48.3
6	RTDETR-FasterNet-CGA-HSFPN	95.8	95.2	85.8	96.7	16,101,128	24.2	47.8
7	RTDETR-FasterNet-CGA-HSFPN_SFF	96.1	96.4	87.4	96.9	16,314,816	24.1	47.9
8	RTDETR-FasterNet-CGA-HSFPN_SFF-CIoU	95.8	96.1	87.3	97.0	16,307,482	24.1	47.8
9	RTDETR-FasterNet-CGA-HSFPN_SFF-Focaler-CIoU	96.4	96.7	89.1	97.2	16,265,580	24.1	47.8

In conclusion, compared to RT-DETR-R18, the proposed FCHF-DETR demonstrates a 1.7% increase in Precision, a 3.1% increase in Recall, a 6% increase in mAP50-95, and a 1% increase in mAP50. The number of parameters decreased by 3.6M, FPS increased by 2.2, FLOP decreased by 9.2G, thereby significantly improving the speed and accuracy of tomato leaf disease detection. Therefore, FCHF-DETR is highly suitable for deployment on terminal devices in agricultural environments, such as cameras, offering the high detection performance necessary for real-world applications.

### 3.4 Visual display

Across a test set comprising 3147 images, FCHF-DETR precisely identified eight types of tomato leaf diseases, alongside healthy leaves, attaining an overall mAP50-95 of 89.1% and an mAP50 of 97.2%.

**Figure 10:**
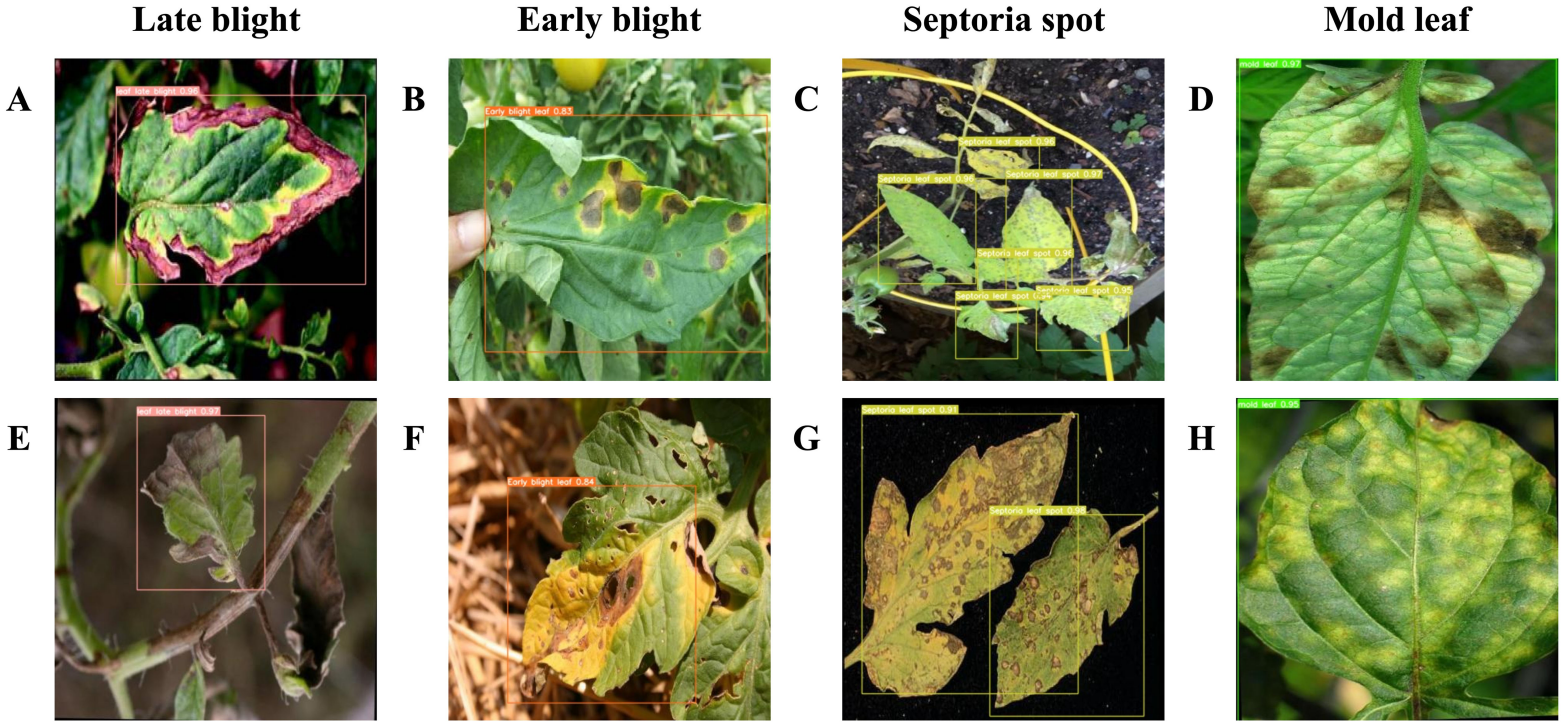
**(A–H)** demonstrate the detection of four distinct types of plant leaf diseases under controlled conditions. The bounding box within the figure highlights the location and specific types of tomato leaf diseases.

To illustrate the detection performance benefits of the proposed method, a visual representation of the detection results for tomato leaf diseases under various conditions is provided. **Figure 10** depicts the model’s detection capability in straightforward settings, characterized by favorable shooting conditions, a simple background, clearly visible affected areas on the tomato leaves, and a minimal number of leaves in the image. **Figures 10A–H** demonstrate the model’s ability to concurrently and accurately detect four distinct tomato leaf diseases in uncomplicated environments: late blight, early blight, Septoria spot, and mold leaf. Given that yellow viruses typically cluster and are found in complex settings, their detection results were not showcased in the depiction of simple environments.

**Figure 11:**
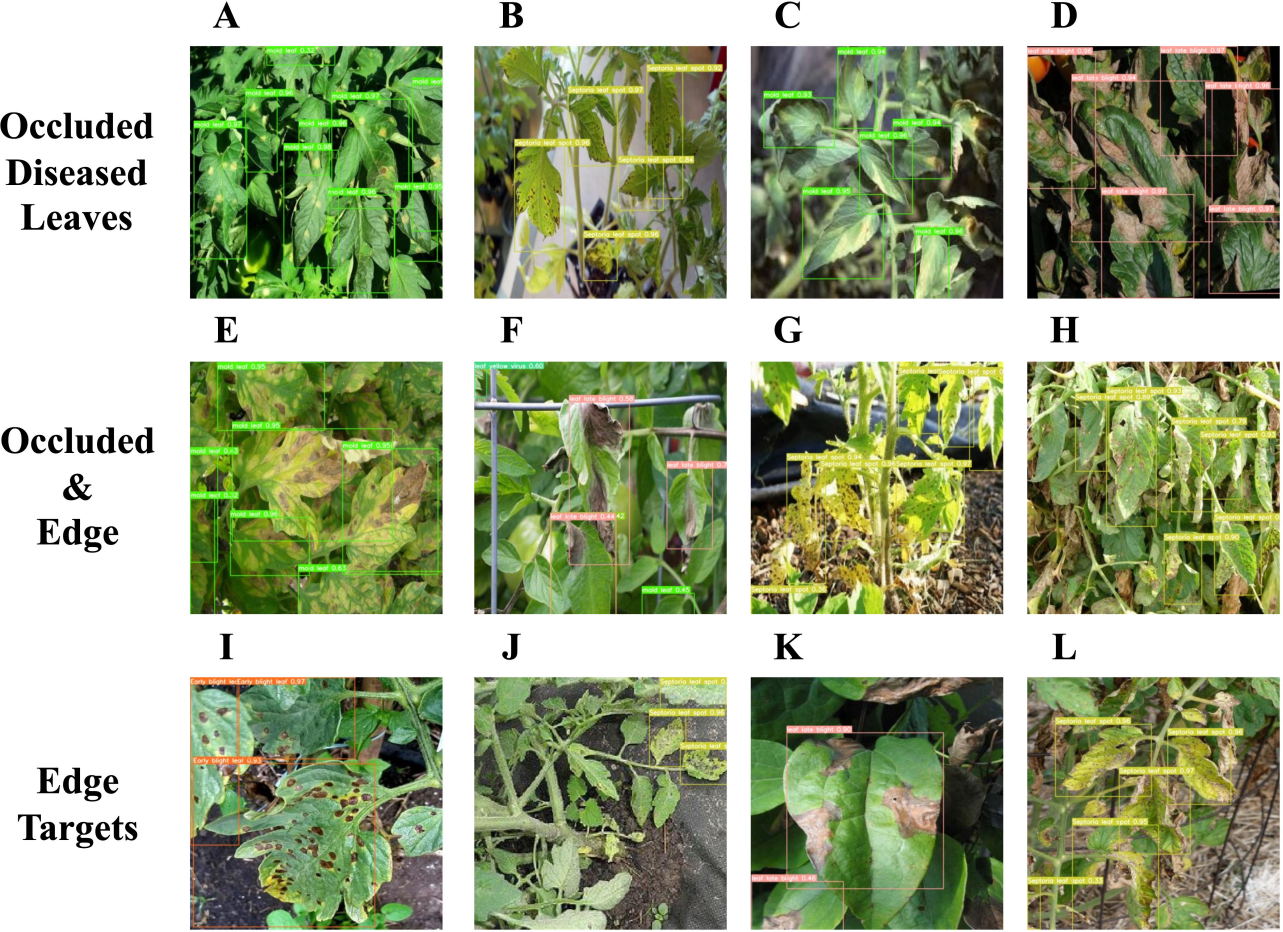
**(A–D)** illustrate the detection performance of the targeted leaf disease in scenarios where it is obscured by other leaves. **(E–H)** demonstrate the detection performance of the targeted leaf disease when situated at the periphery of the image and simultaneously obscured by other foliage. **(I–L)** reveal the detection performance of the targeted leaf disease at the image’s edge.

The integration of the CGA attention mechanism and HSFPN feature fusion module endows the model with a robust capability to extract pivotal information from images, ensuring high detection accuracy across various tomato leaf diseases. **Figure 11** illustrates the model’s detection performance in complex scenarios, including situations where leaves are at the image’s edge or partially obscured. **Figures 11A–D** reveal that the FCHF-DETR model precisely identifies occluded diseased leaves. **Figures 11I–L** demonstrate that, with the Focaler-CIoU loss function integrated, the model enhances the detection accuracy of challenging edge targets, mitigating the original model’s limitation in identifying partially visible diseased leaves. In the other images, the enhanced model is shown to effectively identify edge targets, even those obscured by surrounding foliage.

**Figure 12:**
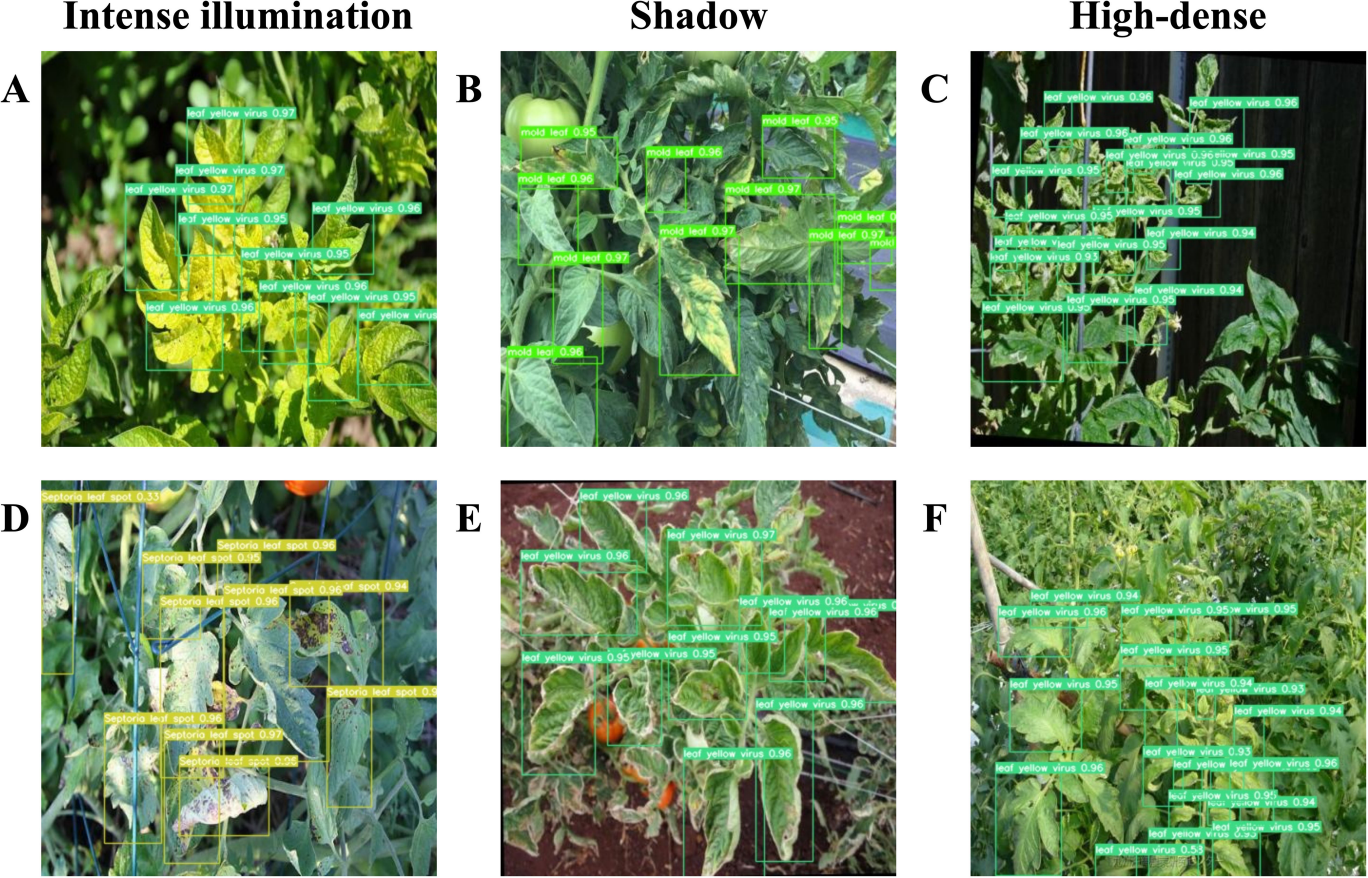
**(A, D)** present the detection results of leaf disease under conditions of intense illumination. **(B, E)** depict the detection results of leaf disease within shaded environments. **(C, F)** illustrate the detection effectiveness of leaf disease in highly dense settings.

To underscore the strengths of the proposed model in complex scenarios, **Figure 12** illustrates its detection capabilities in densely populated environments. Given the dense distribution and potential for small spots on tomato leaves in real-world settings, detecting diseased leaves in such environments is paramount. Despite these challenges, the model maintains robust performance. **Figure 12** demonstrates the model’s efficacy in identifying diseased tomato leaf areas within dense foliage, under varied conditions such as intense illumination area A, D, shadow area B, E, or high-dense area C, F.

**Figure 13:**
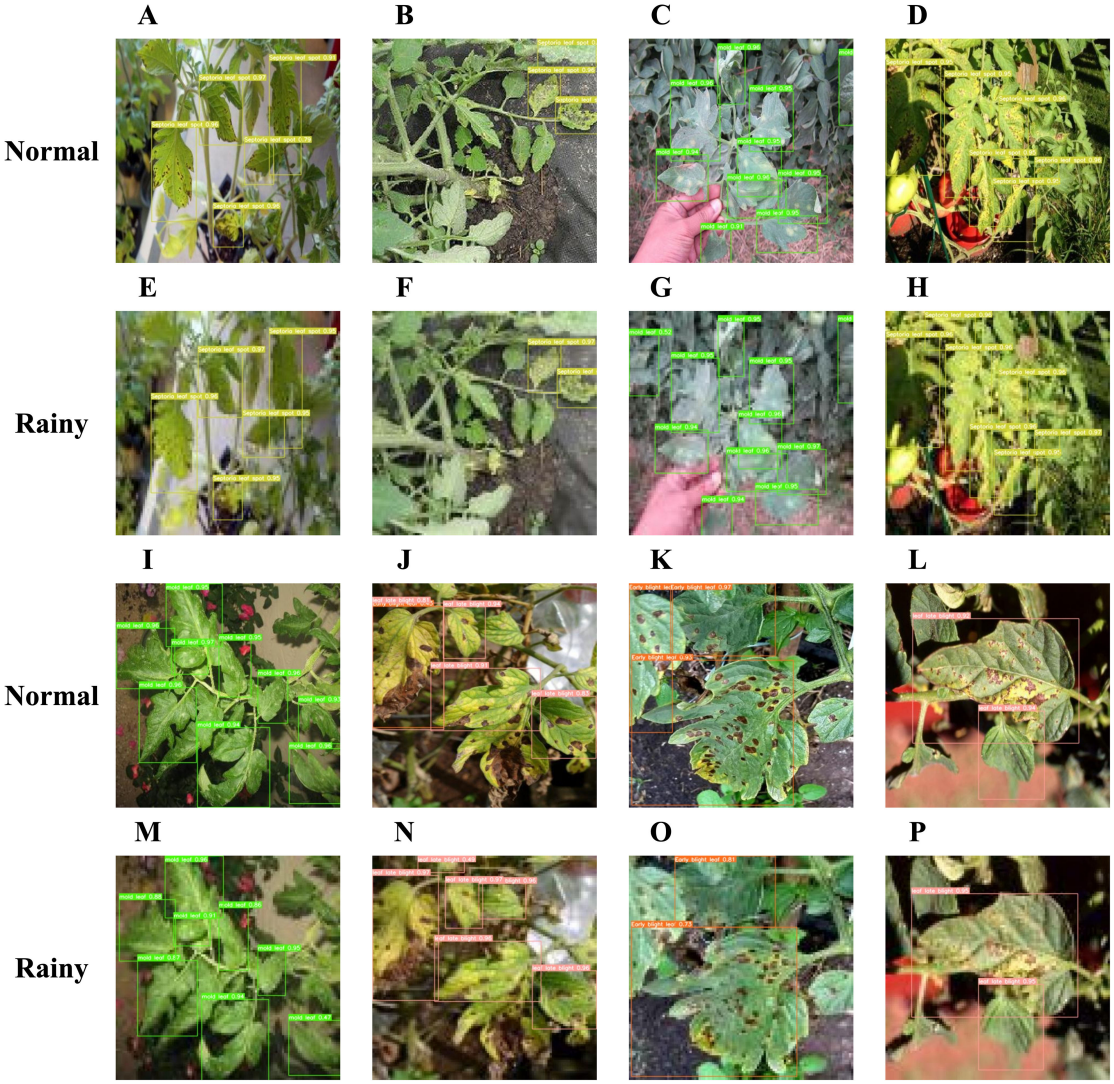
**(A–D)** and **(I–L)** demonstrate the detection efficacy of tomato leaf disease in standard conditions, while **(E–H)** and **(M–P)** exhibit the comparative detection efficacy of the model on the test set following pixel reduction processing and the simulation of rainy conditions within an authentic plantation setting.

Acknowledging weather-related challenges at tomato cultivation sites, pixel reduction was applied to part of the test set data to simulate the effects of rain or dense fog on camera imagery. **Figure 13** reveals that, even with reduced pixel quality, the FCHF-DETR model reliably detects most tomato leaf diseases, with only a minor impact on detection accuracy. The sustained performance in simulated rainy and foggy conditions is credited to the Cascaded Group Attention and HSFPN feature fusion mechanisms within the Efficient Hybrid Encoder, capable of extracting key features from blurred images. Additionally, the incorporation of the Focaler-CIoU loss function enables the detection of leaf diseases that pose challenges for the RT-DETR-R18 model, significantly aiding practical deployment.

The visual evidence from **Figures 10**–**13** confirms that FCHF-DETR adeptly addresses a range of challenges typical in real agricultural settings for tomato leaf disease detection, effectively resolving longstanding issues in the sector.

## 4 Discussion

In contemporary agricultural practices, numerous tomato plants are afflicted by leaf diseases, making manual detection excessively time-consuming and labor-intensive. Current technologies frequently fail to balance processing speed with detection accuracy, particularly when identifying small disease spots, presenting clear drawbacks. To address this challenge, this study introduced FCHF-DETR, a high-precision, lightweight detection algorithm derived from the RT-DETR-R18 framework. A dataset comprising 3147 images of tomato leaf diseases was compiled, encompassing diverse scenes and levels of image clarity. To streamline the model and enhance memory efficiency, the traditional ResNet18 was substituted with FasterNet in the backbone network. Concurrently, within efficient hybrid encoders, replacing the AIFI module with a cascaded group attention mechanism and the CCFM module with HSFPN notably boosted detection accuracy with minimal impact on speed.

Furthermore, to better identify challenging samples, the Focaler-CIoU loss function was introduced, enhancing the model’s performance across the dataset. Experimental results indicated that FCHF-DETR achieved an mAP50-95 of 89.1% on the test set, marking a 6% improvement, and an mAP50 of 97.2%, a 1% increase. Concurrently, FLOPs decreased by 9.2G, and the model’s parameter count was reduced by 3.6M. These achievements showcase the method’s enhancement of detection accuracy and successful reduction in the model’s computational load, illustrating an effective balance between accuracy and efficiency.

In practical agricultural settings, particularly on diverse farmlands, a common challenge arises: the overlapping or obstruction of leaves from different crops, markedly impacting tomato leaf disease detection. For instance, in fields where tomatoes coexist with taller crops like corn or legumes, the foliage of these crops can obscure tomato leaves, masking critical disease features. Under these conditions, the effectiveness of even high-precision detection algorithms like FCHF-DETR may be markedly limited. Leaf occlusion not only diminishes the available feature information for algorithmic recognition but can also lead to errors, like mistaking occluded edges or shadows for disease spots.

This issue underscores the limitations of current visual-based object detection algorithms in navigating complex agricultural scenes. Addressing this challenge necessitates a deeper comprehension of crop interactions and growth patterns to develop algorithms capable of adapting to such diversity and complexity. Furthermore, employing multiperspective or multimodal data acquisition techniques, like integrating aerial and lateral imagery or additional sensor data, could mitigate these issues and enhance lesion detection in occluded conditions.

Meanwhile, we also investigated that the manifestation of tomato leaf disease may vary in different natural environments due to various factors such as climate, soil type, and humidity, resulting in certain types of leaf diseases being more common in specific environments. For example, in high humidity and warm environments, the incidence of downy mildew may be much higher than that of early or late blight in arid environments. The impact of these environmental factors on disease occurrence requires the detection system to adjust the weight of various leaf disease detection according to different natural conditions, in order to improve the detection accuracy and efficiency in specific environments. However, even the high-precision and high-efficiency detection algorithm FCHF-DETR invented in this article adopts the same detection strategy for all types of leaf diseases, failing to fully consider the diversity of natural environmental factors. This may lead to insufficient sensitivity of algorithms to detecting high-risk diseases in certain specific environments, thereby reducing overall detection efficiency and accuracy.

In order to solve this problem, future detection algorithms need to introduce environmental awareness mechanisms, analyze and learn the occurrence patterns of diseases under different natural environmental conditions, and dynamically adjust the detection weights for different leaf diseases. This may involve complex data collection and analysis, such as combining meteorological data, soil conditions, and crop growth data, using machine learning algorithms to predict the probability of disease occurrence under different environmental conditions, and optimizing the parameters of the detection model accordingly. Through this approach, the detection system can adapt more intelligently to different natural environments, improve the detection accuracy of key diseases, and provide more reliable technical support for agricultural production.

## 5 Conclusion

This study introduces FCHF-DETR, a lightweight model for detecting tomato leaf diseases, effectively balancing accuracy and speed. It employs data augmentation and reduction techniques to adapt to real-world environments for detecting tomato leaf diseases. FCHF-DETR enhances the RT-DETR-R18 framework by integrating the lightweight FasterNet backbone, boosting detection speed and reducing model parameters without compromising accuracy. Additionally, it introduces the Cascaded Group Attention mechanism, replacing the AIFI module, and substitutes the CCFM module with HSFPN in the original network. Despite a minor increase in computational speed and model parameters, there’s a significant enhancement in detection accuracy. The adoption of the Focaler-CIOU loss function, replacing the original, further refines the accuracy for challenging samples without altering parameters or computational complexity. Experimental results reveal that FCHF-DETR surpasses RT-DETR-R18 with a 1.7% increase in precision, 3.1% in recall, 1% in mAP50 and 6% in mAP50-95, and reductions in parameters, FPS, and FLOPs. This signifies not just a notable boost in accuracy but also a substantial decrease in the model’s parameter count, thus offering robust support for contemporary tomato leaf disease detection.

Future research will aim to refine detection accuracy in diverse farmlands affected by overlapping leaf occlusion. We plan to leverage multiperspective or multimodal data to develop more adaptive detection algorithms. Additionally, to accommodate varying tomato leaf disease patterns across different environments, future algorithms will incorporate environmental awareness mechanisms. Dynamic adjustments to the detection priorities of different diseases will enhance the accuracy and efficiency in specific environments, broadening the algorithm’s applicability in complex real-world scenarios.

## Data Availability

The original contributions presented in the study are included in the article/supplementary material. Further inquiries can be directed to the corresponding author.
